# Enhancement of vitamin B_6_ production driven by omics analysis combined with fermentation optimization

**DOI:** 10.1186/s12934-024-02405-1

**Published:** 2024-05-15

**Authors:** Zhizhong Tian, Linxia Liu, Lijuan Wu, Zixuan Yang, Yahui Zhang, Liping Du, Dawei Zhang

**Affiliations:** 1 College of Biotechnology, Tianjin University of Science and Technology, Tianjin, 300457 China; 2 Tianjin Institute of Industrial Biotechnology, Chinese Academy of Sciences, Tianjin, 300308 China; 3 National Center of Technology Innovation for Synthetic Biology, Tianjin, 300308 China; 4 Key Laboratory of Engineering Biology for Low-Carbon Manufacturing, Tianjin Institute of Industrial Biotechnology, Chinese Academy of Sciences, Tianjin, 300308 China; 5 University of Chinese Academy of Sciences, Beijing, 100049 China

**Keywords:** Vitamin B_6_, Omics analysis, Cellular processes, Fermentation optimization

## Abstract

**Background:**

Microbial engineering aims to enhance the ability of bacteria to produce valuable products, including vitamin B_6_ for various applications. Numerous microorganisms naturally produce vitamin B_6_, yet the metabolic pathways involved are rigorously controlled. This regulation by the accumulation of vitamin B_6_ poses a challenge in constructing an efficient cell factory.

**Results:**

In this study, we conducted transcriptome and metabolome analyses to investigate the effects of the accumulation of pyridoxine, which is the major commercial form of vitamin B_6_, on cellular processes in *Escherichia coli*. Our omics analysis revealed associations between pyridoxine and amino acids, as well as the tricarboxylic acid (TCA) cycle. Based on these findings, we identified potential targets for fermentation optimization, including succinate, amino acids, and the carbon-to-nitrogen (C/N) ratio. Through targeted modifications, we achieved pyridoxine titers of approximately 514 mg/L in shake flasks and 1.95 g/L in fed-batch fermentation.

**Conclusion:**

Our results provide insights into pyridoxine biosynthesis within the cellular metabolic network for the first time. Our comprehensive analysis revealed that the fermentation process resulted in a remarkable final yield of 1.95 g/L pyridoxine, the highest reported yield to date. This work lays a foundation for the green industrial production of vitamin B_6_ in the future.

**Supplementary Information:**

The online version contains supplementary material available at 10.1186/s12934-024-02405-1.

## Background

Low-carbon and sustainable manufacturing has emerged as a topical subject in global economic development [1, 2]. In particular, environmentally friendly microbial manufacturing has experienced rapid growth across various fields of food, medicine [3], and energy, which has brought significant economic benefits and generated substantial social value worldwide [4]. Recent advances have been made in many commercial cases of microbial manufacturing using high-performance strains in trans-aconitic acid [5], lipoic acid [6], cinnamaldehyde [7], L-leucine [8] and other products [9]. Vitamin B_6_ encompasses a group of vitamins, namely, pyridoxal (PL), pyridoxine (PN), and pyridoxamine (PM), along with their corresponding phosphate esters, 5’-pyridoxal phosphate (PLP), 5’-pyridoxine phosphate (PNP), and 5’-pyridoxamine phosphate (PMP) [9–11]. Vitamin B_6_, also known as VB_6_, plays a crucial role as a cofactor for numerous proteins and enzymes, making it one of the most significant vitamins [12, 13]. Most enzymes that rely on pyridoxal phosphate (PLP) as a cofactor [14] are involved in various biochemical processes, such as amino acid biosynthesis, decarboxylation, racemic reactions, Cα-Cβ bond cleavage, elimination, and the replacement of α, β, and γ [15]. The enzymes involved in the pathway of deoxysugar biosynthesis utilize pyridoxamine phosphate (PMP) as a cofactor. Among the various production forms, PN holds the utmost significance [16]. Vitamin B_6_ is a valuable compound for the pharmaceutical industry [9], food and feed additives [17], and cosmetics [18]. Recently, it has been reported that engineered *Escherichia coli* (*E. coli*) can produce 1.4 g/L PN by decoupling the de novo synthesis pathway, protein engineering of key enzymes, and using a multimodule iterative optimization strategy, which is the highest PN yield ever reported [16]. However, the intrinsic connection between cell metabolism and culture conditions is not clear, and optimization methods are needed to explore to further enhance PN production [19].

With the rapid development of omics technologies, the process optimization of microbial manufacturing can also be guided by omics, including genomics, transcriptomics, proteomics, metabolomics etc [1]. Metabolomics, transcriptomics and proteomics [20] approaches can detect changes in intracellular metabolism and thus help improve the efficiency of product synthesis pathways [21]. RNA-seq is mainly applied to study transcriptomic differences caused by various treatments [22]. The overall analysis of transcription, translation and metabolism at the molecular level allowed the identification of key differentially expressed genes (DEGs) and provided new insights into the molecular mechanisms involved in target molecule production [23]. Zhu et al. [24] used RNA-Seq to explore the effects of nitrogen sources and ICDH (isocitrate dehydrogenase) knockout on glycolate production in *E. coli*. Transcriptome analysis under different fermentation conditions was performed, and the significantly altered genes related to N regulation, the oxidative stress response, and iron transport were analyzed. The latter strain, Mgly624 (with the ICDH deletion in Mgly534), achieved a balance between cell growth and glycolate production, reaching a glycolate production of 0.81 g glycolate/OD, which was 2.6-fold greater than that of the previous strain Mgly534. Notably, RNA-seq significantly contributed to revealing the importance of the ICDH gene. Liang et al [25]. used RNA-Seq technology to analyze the gene expression of two strains, *Pseudomonas balearica* R90 and *Brevundimonas diminuta* R79, during cocultivation. The expression of genes related to the synthesis of polyhydroxyalkanoate (PHA), specifically the *acs* (encoding acetyl-CoA synthetase) and *phaA* (encoding acetyl-CoA acetyltransferase) genes, was upregulated in the coculture group compared to the pure culture groups. This enhanced the utilization of acetic acid and the synthesis of poly-β-hydroxybutyrate (PHB), leading to a considerably greater yield of PHB in the coculture group than in the pure culture group. Li et al [26]. used RNA-seq technology to investigate the coproduction mechanism of poly-γ-glutamic acid (γ-PGA) and nattokinase in *Bacillus subtilis* natto. In this study, gene expression at different fermentation periods (6 h, 9 h, and 24 h) was analyzed, and the key genes involved in the production of γ-PGA and nattokinase were identified. By analyzing the main metabolic pathways, potential target genes for enhancing nattokinase activity and γ-PGA yield were also identified. The maximum γ-PGA yield obtained was 358.5 g/kg sucrose when 50 g sucrose was added per kilogram of substrate. The authors also observed the upregulation of genes related to glutamate synthesis and the downregulation of γ-PGA-degrading enzymes, which could contribute to an increase in yield. Thus, with RNA-Seq, we can study global transcriptional changes and gain new insights into strains that produce PN.

In the present study, we aimed to better understand the implications of artificially altering the metabolic pathways of the strains by transcriptomic profiling and metabolomics. This includes studying the various changes that may occur, such as changes in glycolysis, the tricarboxylic acid (TCA) cycle, amino acids, cofactors, and the response to fermentation conditions. Here, we revealed the metabolic network changes behind the PN production and provided clues for improving PN production via fermentation optimization.

## Results and discussion

### Characterization of pyridoxine produced by engineered *E. coli*

In a previous study, we constructed the PN high-production strain LL388 [16]. This strain was transformed with two plasmids, one containing the backbone of pRSFDuet-1, and the other containing p15ASI. pRSFDuet-1 carries mutated genes from *E. coli*, including *pdxA2* (encoding 4-hydroxythreonine-4-phosphate dehydrogenase) and *pdxJ1* (encoding pyridoxine 5-phosphate synthase). p15ASI carries *epd* (encoding D-erythrose 4-phosphate dehydrogenase) from *Glaciecola nitratireducens*, *pdxB* (a native gene encoding erythronate-4-phosphate dehydrogenase), *dxs* (encoding 1-deoxy-D-xylulose-5-phosphate synthase) from *Ensifer meliloti*, and *serC* (a native gene encoding phosphoserine aminotransferase). These genes encode all the enzymes involved in the PN biosynthesis process, and we rationally modified and/or heterologously screened these genes in the previous study [16]. The PN yield of the strain reached 450 mg/L in the shake flask and 1.4 g/L in 5 L of fed-batch fermentation, but there was fermentation heterogeneity owing to metabolic burden and plasmid stability. It characterized that PN production was coupled with cell growth. However, many of our fed-batch experiments showed that cell growth arrest hinders PN production. In bioreactors, the OD_600_ value representing cell growth is usually approximately 30–40, which greatly limits the increase in PN production. To identify potential gene targets for improving PN production and find the factors that stop cell growth, transcriptome profiling was conducted to identify changes in global gene transcription levels in response to PN [27–29]. Transcriptome analysis is an effective technique for studying genome-wide gene expression changes in microorganisms and has been widely applied to uncover potential genes and elucidate the molecular mechanisms underlying various metabolic processes [30–32].

In this study, transcriptome samples were obtained from the LL388 strain in fed-batch fermentation cultured at 37 °C and pH 6.8. The glycerol-containing solution started to feed at ∼ 10 h when the initial glycerol concentration was less than 3 g/L during fermentation, and the initial glycerol concentration was 15 g/L. From 6 to 7 h, the cells undergo a transition from the lag phase and enter a phase of rapid growth, and the production of PN is coupled to cell growth. As a result, the cells of LL388 were collected, and comparative analysis of their transcriptional profiles was conducted at 6 and 16 h, corresponding to the mid-to-late log phase (Fig. S1). In addition, the main byproducts we assayed included acetate, lactate, pyruvate, succinate, α-ketoglutaric acid, citric acid, etc., but the accumulation of these organic acids was not detected. Therefore, it may not be that the byproducts in the supernatants repressed cell growth, so we performed intracellular metabolomic analysis to further identify the bottleneck of PN production at 6, 26, 36, and 42 h.

### Overall analysis of the response of DEGs to the production enhancement mechanism of pyridoxine

**Fig. 1:**
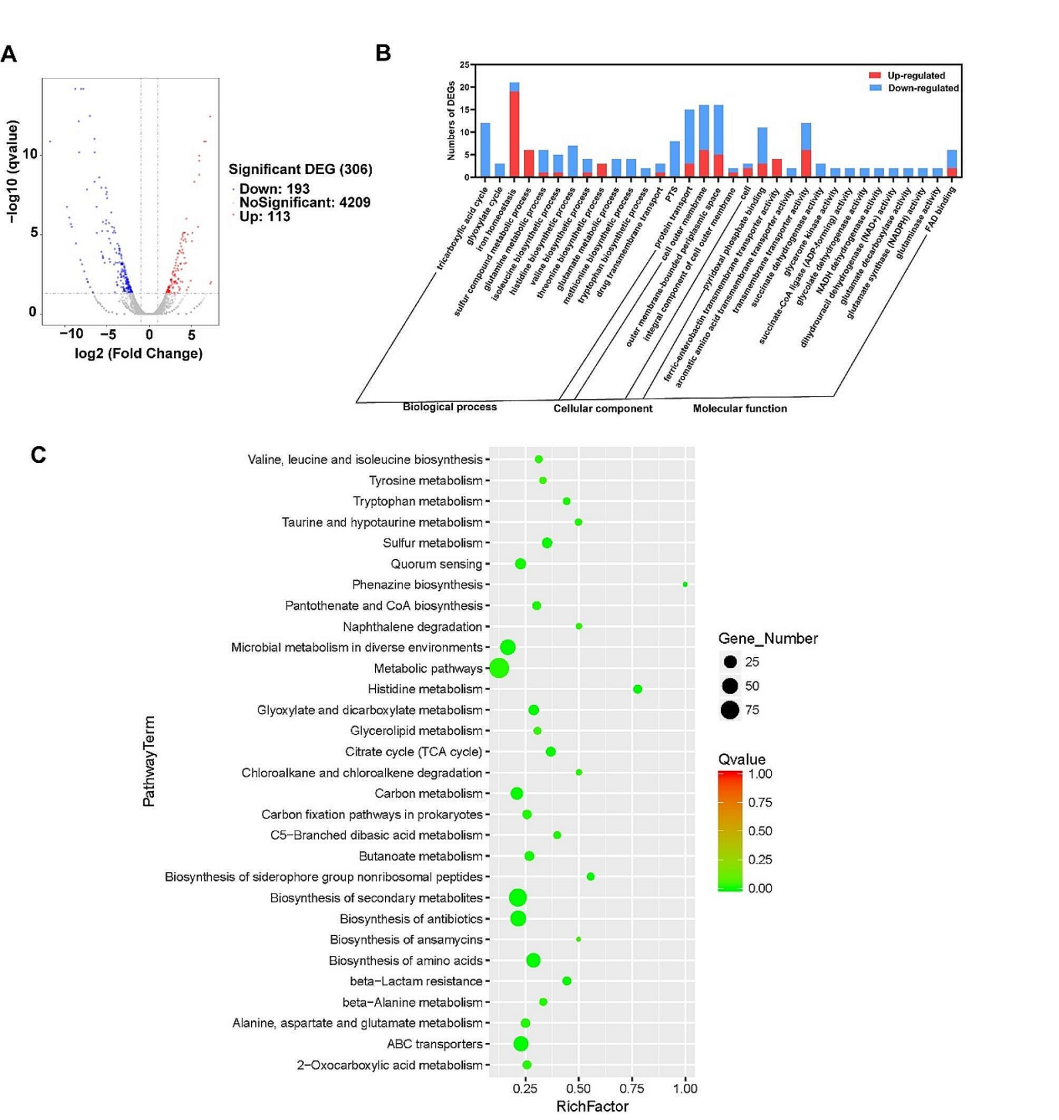
Gene Ontology (GO) and pathway enrichment analysis. **(A)** Volcano plot depicting the transcriptome data as a relation between the q value and fold change. **(B)** GO term analysis of the DEGs showing three aspects: molecular function, cellular component and biological process. **(C)** Pathway enrichment analysis of the DEGs.

Comparative transcriptome analyses were performed during the log phase at 6 h and 16 h, when the OD_600_ increased from 9.43 to 29.3 and the PN titer increased from 98.0 to 262.2 mg/L (Fig. S1). The transcript levels of 306 genes significantly changed (log2|FoldChange|≥ 2.0 and FDR ≤ 0.05) upon PN accumulation, 193 of which were significantly downregulated and 113 of which were significantly upregulated (Fig. 1A). The differentially expressed genes (DEGs) were then subjected to Gene Ontology (GO) term enrichment analysis, and Kyoto Encyclopedia of Genes and Genomes (KEGG) pathway enrichment analysis was subsequently conducted to identify the pathways associated with the genes whose expression significantly increased.

In these paired samples at different points, GO functional enrichment analysis was carried out. Notably, “TCA cycle”, “iron assimilation, homeostasis and transport”, and “amino acid metabolic process” were the dominant terms in the “biological process”. In addition, we also identified a relatively large number of genes associated with transmembrane transport (Fig. 1B). GO functional enrichment analysis revealed that the largest number of downregulated genes were enriched in the TCA cycle, carbon metabolism, amino acid biosynthesis and transport, while the upregulated genes were enriched in the processes of iron assimilation, homeostasis and transport. Additionally, DEGs were mapped to 32 KEGG pathways, and the significantly enriched pathways are shown in Fig. 1C. The KEGG analysis revealed that the primary enriched genes were associated with amino acid biosynthesis or metabolism, the TCA cycle, carbohydrate metabolism, etc.

**Fig. 2:**
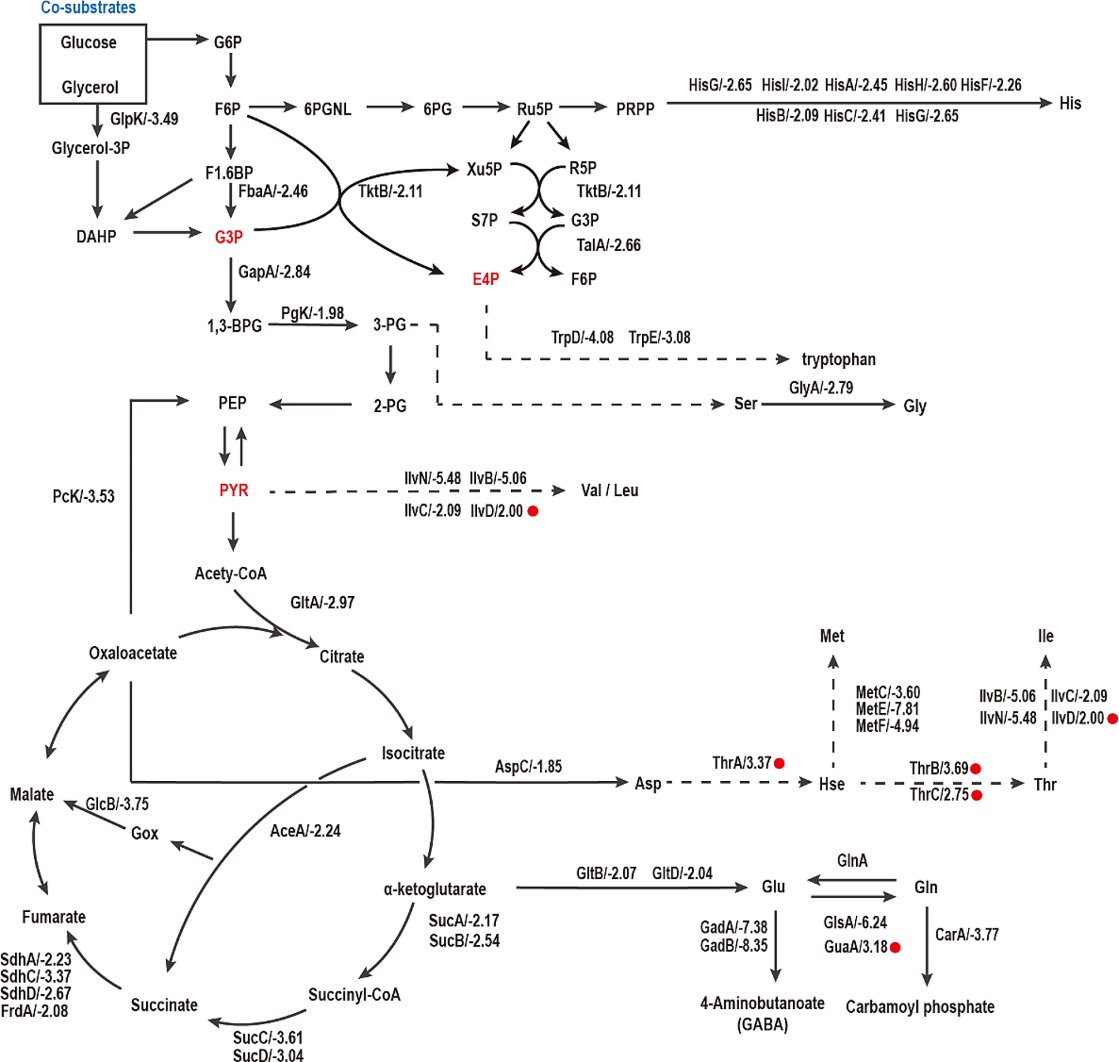
Transcriptome analysis of the mutant strain *E. coli* in response to PN overproduction from 6 h to 16 h. The red font represents the precursors of PN, and the red dots indicate the upregulated genes. The numbers after the gene showed the fold change in the transcriptome analysis

Glycolysis, the TCA cycle and the electron transport chain are ubiquitous and important pathways in *E. coli* [33, 34]. Detailed analyses revealed that most genes related to glycolysis and the TCA cycle were downregulated, especially genes related to the TCA cycle (Fig. 2). This observation suggested that PN accumulation may inhibit central carbon metabolism, subsequently leading to a decrease in PN production in the engineered strain. This is in line with the observed reduction in the growth curve. Amino acid biosynthesis or metabolism was another significant category of genes downregulated in response to PN accumulation (Fig. 2). The downregulated genes were involved in the biosynthesis or metabolism of glutamate, L-aspartate, L-histidine, tryptophan, L-valine/L-isoleucine/L-leucine, the aspartate family, etc. Combined with the downregulation of glycolysis and the TCA cycle, we inferred that the accumulation of PN might inhibit cell growth through its impact on the synthesis of related amino acids. A previous study reported that 4-hydroxy-threonine (4HT), a branched intermediate of the native vitamin B_6_ pathway, has significant negative effects on the biosynthesis of the aspartate-derived amino acid threonine and the branched-chain amino acids isoleucine and leucine [35]. Additionally, 4HTP is a classical competitive inhibitor of the threonine synthase ThrC from *E. coli*. Consistently, we found that the transcription of genes involved in isoleucine biosynthesis pathway, such as *ilvN*, *ilvB*, and *ilvC*, was largely repressed by PN accumulation (Fig. 2). The transcription of *thrC* was upregulated, while the corresponding enzyme can catalyze 4HTP to form 4HT [36]. It is reasonable that 4HTP is shunted to form 4HT, both of which inhibit the biosynthesis of some amino acids [9]. 4HT is a competitive inhibitor of ThrB that affects the conversion of homoserine to O-phospho-L-homoserine. This inhibition leads to the suppression of the synthesis of amino acids such as threonine and isoleucine [35]. Additionally, the downregulation of histidine biosynthetic genes may be caused by the induction of R5P, which provides PLP for cell growth and PN production [37].

**Fig. 3:**
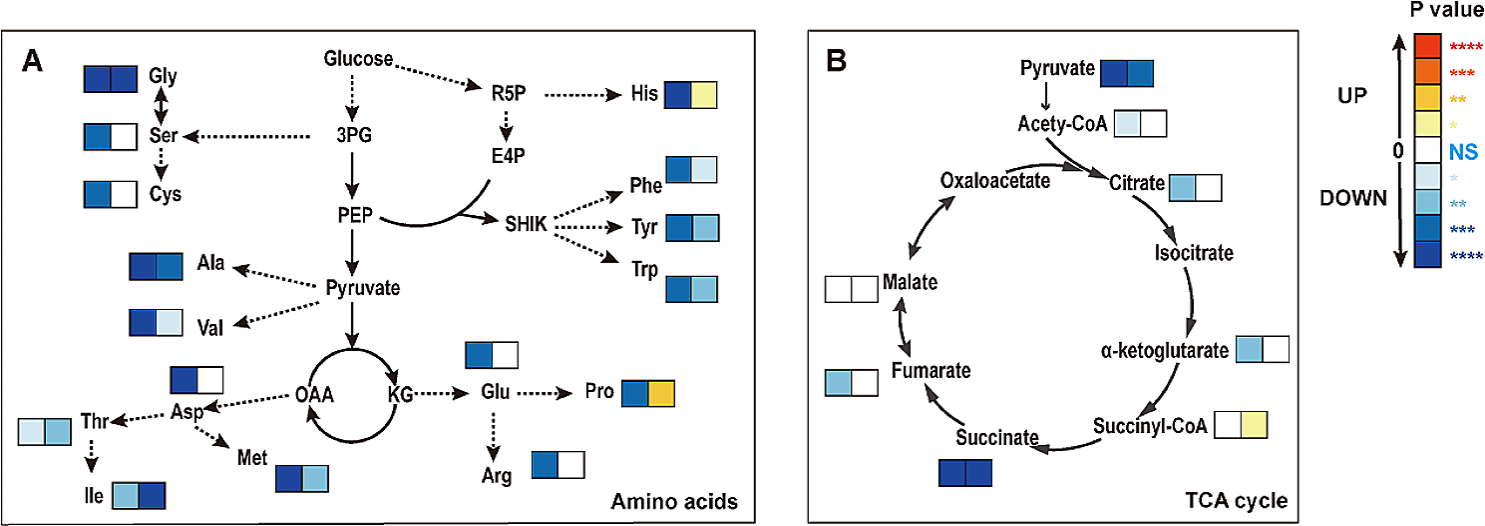
Metabolic analysis of the mutant strain *E. coli* in response to PN overproduction between the early and late stages of fermentation. **(A)** Amino acid analysis. **(B)** Chemical analysis of the TCA cycle. Among the two adjacent squares, the squares on the left represent changes occurring in the early phase (from 6 to 16 h), while the squares on the right represent changes occurring in the late phases (from 36 to 42 h). The experiments were conducted in triplicate, and the significance (*p* value) was evaluated by two-sided Student’s *t* test. * *P* < 0.05; ** *P* < 0.01; ***P < 0.001; **** *P* < 0.0001

Furthermore, metabolic alterations were investigated by untargeted metabolomic profiling analysis via ultra-performance liquid chromatography‒mass spectrometry (UPLC-MS). Samples at different time points (6, 16, 36, and 42 h) were selected for metabolomics analysis. For the metabolome, |Error(ppm)| ≤10 and *P* value were used to screen for differentially expressed metabolites. Metabolic pathway analysis was based on the KEGG database. In this study, we identified 17 metabolites involved in amino acid biosynthesis and 10 metabolites involved in the TCA cycle. The levels of amino acids, such as histidine, aspartate, and valine, were significantly reduced from 6 to 16 h (early stage of fermentation) but showed a variable trend from 36 to 42 h (late stage of fermentation) (Fig. 3). For instance, there was a substantial increase in the level of histidine during the late stage of fermentation, whereas the level of valine decreased markedly. No noticeable change was observed in the aspartate level. These adjustments reflect the series of changes that bacteria undergo to adapt to their environment. The results were consistent with the transcriptome analysis. The precursor for PN biosynthesis in *E. coli* is DXP (1-deoxy-D-xylulose-5-phosphate), which can be synthesized from pyruvic acid and G3P through Dxs (1-deoxy-D-xylulose-5-phosphate synthase). The relative abundance of pyruvic acid dramatically decreased during PN production, indicating that PN production significantly increased the consumption of pyruvate. Additionally, succinate was significantly decreased in the TCA cycle, and less α-ketoglutarate (α-KG) was produced, especially during early fermentation. These metabolomic results are consistent with the transcriptomic data, indicating that PN production might inhibit the synthesis of succinate and disturb amino acid metabolism. These results could provide insight into the relationship between metabolite levels and gene expression during PN production.

### Construction of a single plasmid-engineered strain

Based on the findings from transcriptome analysis and metabolic profiling, a hypothetical model of the mechanism of PN inhibition has been proposed. This model suggests that the disruption of the TCA cycle, particularly the impaired production of succinate, as well as the partial synthesis of amino acids, collectively contributes to the inhibition of cell growth and the accumulation of PN. To further reduce the effects of PN inhibition or 4HTP toxicity and enhance final PN production, medium component optimization, especially nitrogen optimization, was performed to improve the growth of *E. coli* cells.

**Fig. 4:**
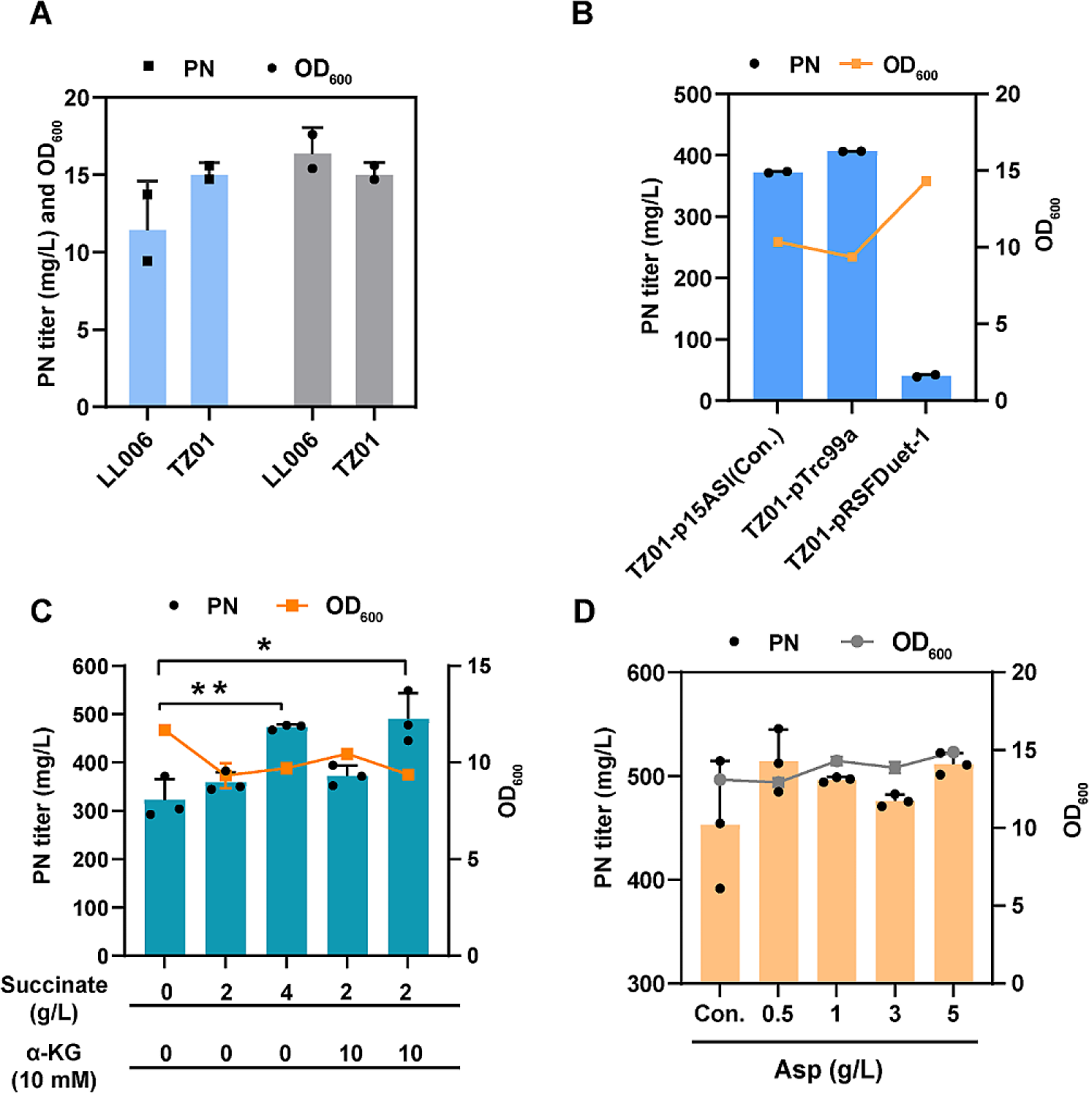
Construction of the chassis cell and fermentation optimization. **(A)** PN titer and cell growth (OD_600_) of TZ01 with one copy inserted into the genome at the *rpnD* locus. **(B)** PN titer and cell growth (OD_600_) of TZ01 transformed with different plasmids. **(C)** PN titer and cell growth (OD_600_) of TZ03 after the addition of succinate and α-KG. The experiments were conducted in triplicate, and the significance (*p* value) was evaluated by two-sided Student’s *t* test. **P = 0.0038, *P = 0.0131. (D) PN titer and cell growth (OD_600_) of TZ03 after the addition of different concentrations of Asp

Initially, to increase PN production, we integrated two copies of the *pdxA2-pdxJ1* operons into the LL006 genome and created a single plasmid to reduce the instability associated with using two plasmids. The newly obtained strain was named TZ01, and its PN production increased (Fig. 4A). The high-copy plasmids pTrc99a and pRSFDuet-1 were optimized as single vector backbones to carry *epd*-*pdxB*-*dxs*-P_J231119_-*serC* fragments [16]. The promoter and ribosome binding sites (RBSs) employed were consistent with those used by p15ASI, specifically the *tac* promoter, to regulate the transcription of *epd*-*pdxB*-*dxs*. Additionally, the *J23119* promoter was used to control the expression of *serC*. The results indicated that pTrc99a (Fig. S2) exhibited greater PN production than pRSFDuet-1 and slightly greater PN production than the original p15ASI plasmid (Fig. 4B). The new strain TZ03 (TZ01 harboring pTrc99a-P_tac_-*epd* (Gni)-*pdxB* (Eco)-*dxs* (Eme)-P_J231119_-*serC* (Eco)) was employed as the chassis cell for fermentation optimization experiments.

### Fermentation optimization of decreased TCA compounds and amino acids

Optimization of the fermentation medium by incorporating cost-effective chemicals has been demonstrated to be an effective approach for enhancing the production of specific target molecules. According to the results of omics analysis, first, we added some significantly downregulated molecules, including succinate, histidine, and threonine, to the fermentation medium FM1.4 to increase cell viability and enhance PN production. The addition of succinate altered the pH, so the concentration added was up to 4 g/L. The concentration of α-KG used was based on previous studies and was 10 mM. The synthesis of PN by the recombinant strain TZ03 was promoted to 490 mg/L by the addition of 2 g/L succinate and 10 mM α-KG (Fig. 4C). The addition of α-KG can enhance PdxB turnover by disrupting the tight binding state between the enzyme and its cofactor NADH [38], which enhances the metabolic flow of the vitamin B_6_ pathway. However, the specific role of succinate in the production of vitamin B_6_ remains unknown. Further research is needed to determine its precise contribution to the production process.

Not all components present in the medium contribute to metabolite production. Therefore, it is of utmost importance to eliminate noncontributing factors from the study as early as possible. To enhance the production of PN, a statistical method of medium optimization was used. Plackett–Burman design (PBD) is a well-established and widely used statistical technique for efficiently screening medium components [39, 40]. It is a two-level design that is very useful for economically detecting the main effects while assuming that all other interactions are negligible [41]. Despite the complexity and numerous interactions within cellular networks, we attempted to identify important amino acid components, including 10 downregulated candidates from the omics analysis. Table 1 represents the Plackett–Burman experimental design for 12 trials with two concentrations for each variable and the corresponding PN activity. The variable represents 10 amino acids; however, the confidence level of the variable is less than 95%, and hence, the variable is considered to be insignificant (Table 1 and Table S1). The above results indicated that a single amino acid had no significant effect on PN production.

**Table 1:** PBD matrix of amino acids with PN production

Trial no.	Variables levels^a^										PN production (mg/L)	
	Gly	His	Val	Asp	Thr	Met	Ile	Glu	Arg	Trp		
1	-	+	+	+	-	+	+	-	+	-	193.37 ± 26.28	
2	+	+	-	+	+	-	+	-	-	-	409.76 ± 2.85	
3	+	+	+	-	+	+	-	+	-	-	389.86 ± 1.14	
4	+	-	+	+	-	+	-	-	-	+	373.52 ± 0	
5	-	-	+	+	+	-	+	+	-	+	359.58 ± 51.58	
6	+	-	-	-	+	+	+	-	+	+	376.72 ± 13.86	
7	-	-	-	-	-	-	-	-	-	-	389.14 ± 16.84	
8	-	+	+	-	+	-	-	-	+	+	391.52 ± 22.16	
9	+	-	+	-	-	-	+	+	+	-	407.86 ± 0	
10	-	-	-	+	+	+	-	+	+	-	382.77 ± 6.60	
11	+	+	-	+	-	-	-	+	+	+	325.73 ± 18.75	
12	-	+	-	-	-	+	+	+	-	+	367.20 ± 3.24	
Confidence level (%)	37.6	39.4	27.2	47.7	44.8	37.6	27.6	20.4	39.2	4.6		

^a^ +, high concentration of variable, 0.6 g/L; -, low concentration of variable, 0.4 g/L

Additionally, yeast extracts and Bacto peptone are rich sources of peptides and mixed amino acids [42]. Optimizing the concentration of yeast extracts and Bacto peptone provides an alternative approach to replenish the diminished amino acid pool [43]. This optimization is closely linked to the carbon to nitrogen (C/N) ratio, which plays a crucial role in fermentation medium [44]. Microorganisms rely on a well-balanced ratio of carbon to nitrogen to sustain their activity [45, 46]. By adjusting the concentration of yeast extracts and Bacto peptone, it becomes possible to modulate the availability of carbon and nitrogen in the fermentation medium [46]. This ensures that microorganisms have an optimal nutrient supply to support their growth and metabolic processes. While optimizing amino acids, we optimized the PN production of glycerol, glucose, yeast extract, and Bacto peptone by orthogonal testing. Orthogonal testing is a method of designing and conducting experiments to test multiple factors and their interactions in a systematic and efficient manner [47]. It involves selecting a set of test cases that are independent and do not overlap in their effects on the system being tested. This helps identify the most important factors and interactions while minimizing the number of test cases needed. The carbon-nitrogen ratio ranged from 3.55 to 9.92 (Table 2). The results showed that the inclusion of an organic nitrogen source resulted in an increase in PN production compared with the original medium, with the optimal combination of glycerol at 12 g/L, glucose at 4 g/L, yeast extract at 8 g/L, and Bacto peptone at 7 g/L. However, it was observed that cell growth was superior to that under other conditions. One possible explanation for these results is that the organic nitrogen source primarily stimulated the growth of the microorganisms, as evidenced by the increased optical density (OD_600_), which affected PN production (Table 2).

**Table 2:** The orthogonal array of different C/N ratios

Num.	Glycerol (g/L)	Glucose (g/L)	YE^b^ (g/L)	Peptone (g/L)	C/N	OD_600_	PN (mg/L)
1	15	1	3	7	7.12	11.5 ± 0.11	347.23 ± 14.36
2	15	4	5	5	8.16	12.65 ± 0.03	383.08 ± 13.10
3	15	6	8	2	8.56	13.46 ± 0.16	372.51 ± 32.97
4	12	1	5	2	7.65	9.91 ± 0.10	316.26 ± 7.77
5	12	4	8	7	4.56	14.95 ± 0.10	406.3 ± 12.84
6	12	6	3	5	9.92	10.49 ± 0.18	229.83 ± 4.20
7	10	1	8	5	3.55	13.61 ± 0.39	279.46 ± 20.40
8	10	4	3	2	11.83	7.51 ± 0.14	148.45 ± 9.34
9	10	6	5	7	5.84	13.37 ± 0.11	342.4 ± 15.57
0^c^	15	1	5	5	6.76	11.27 ± 0.08	319.41 ± 4.10

^b^YE: Yeast extract

^c^The original medium was used as the control

To improve the yield of metabolites, it is necessary to optimize the production medium. Fermentation optimization of specific amino acids and mixed amino acids (such as organic nitrogen sources) in the medium increased the PN yield, but the highest yield was approximately 400 mg/L (409.7 mg/L amino acid pool and 406.3 mg/L organic nitrogen source). To simplify the medium preparation process, we introduced an improved carbon and nitrogen formula known as FM1.5. This upgraded formula was designed to enhance the efficiency of the medium for producing PN. Furthermore, among the ten amino acids, Thr and Asp exhibited higher confidence levels. This suggests that these two amino acids may play a significant role compared to the other amino acids. Specifically, these findings indicate that the toxic compound 4HTP indeed impairs the biosynthesis of Thr, as the addition of Thr can reverse the inhibition caused by 4HTP. Asp, which is upstream of Thr in the biosynthetic pathway (Figs. 2 and 3), is of particular interest due to its downregulation. Based on our analysis, we speculate that the addition of different concentrations of Asp will have a positive effect on the yield of PN. Through the exogenous addition of Asp, we observed a consistent improvement in pyridoxine (PN) production across all tested concentrations, including 0.5, 1, 3, and 5 g/L (Fig. 4D). This finding supports our hypothesis, as the addition of 0.5 g/L Asp resulted in the highest PN titer, reaching 514.30 mg/L.

### Fed-batch fermentation optimization

To assess the feasibility of scaling up PN production using a one-plasmid strain, fed-batch cultivation of strain TZ03 was performed in a 5 L bioreactor. Four fed-batch cultures were conducted, employing media formulations containing succinate as the carbon source and varying concentrations of the nitrogen source. By systematically evaluating these different combinations, the aim was to identify the optimal concentration that would yield the highest PN production.

In the fed-batch cultivation, a mixture of glycerol and glucose was used as the carbon source. Throughout the entire fermentation process, the concentration of glycerol was maintained below 3 g/L. Additionally, we observed that the proportion of glucose was relatively low, and glucose was preferentially consumed over glycerol. The control of low glycerol concentrations helps prevent the excessive accumulation of glycerol, which can hinder the growth and productivity of the strain. The pH of the culture broth was maintained at 6.5 by automatically feeding with 25% (w/v) NH_3_·H_2_O.

In fed batch I, we investigated the production capacity of the single plasmid strain using the original medium supplemented with 4 g/L succinate in the medium in a bioreactor. The dissolved oxygen (DO) level was maintained at 30%. The results indicated that the maximum optical density (OD_600_) reached only 40, indicating the slow growth of the microorganisms. However, at 10 h into fermentation, PN accumulation (∼ 103 mg/L) commenced, and its concentration increased to 1 g/L by 46.5 h. The accumulation of PN rapidly increased throughout the entire fermentation process, particularly during the early to mid-stage of fermentation, concurrent with cell growth (as shown in Fig. S3). Ultimately, the PN productivity was measured to be 1.177 g/L/h after 54 h of fermentation. Various organic acids, including acetate, succinate, pyruvate, formic acid, and citric acid, were tested in the fermentation process. However, only succinate, pyruvate, and acetate were detectable. The results revealed that the amount of succinate rapidly decreased over time, indicating that succinate was consumed during fermentation. This observation aligns with the absence of succinic acid in the previous fermentation process from shaking flasks. On the other hand, the acetate concentration increased throughout the fermentation process, while the pyruvate concentration initially increased and subsequently decreased (Fig. S4). However, we speculated that the minimal accumulation of byproducts such as acetate and pyruvate does not significantly hinder growth. There are likely other crucial factors that truly impact growth, such as succinic acid and the analyzed amino acid pool (organic nitrogen source).

**Fig. 5:**
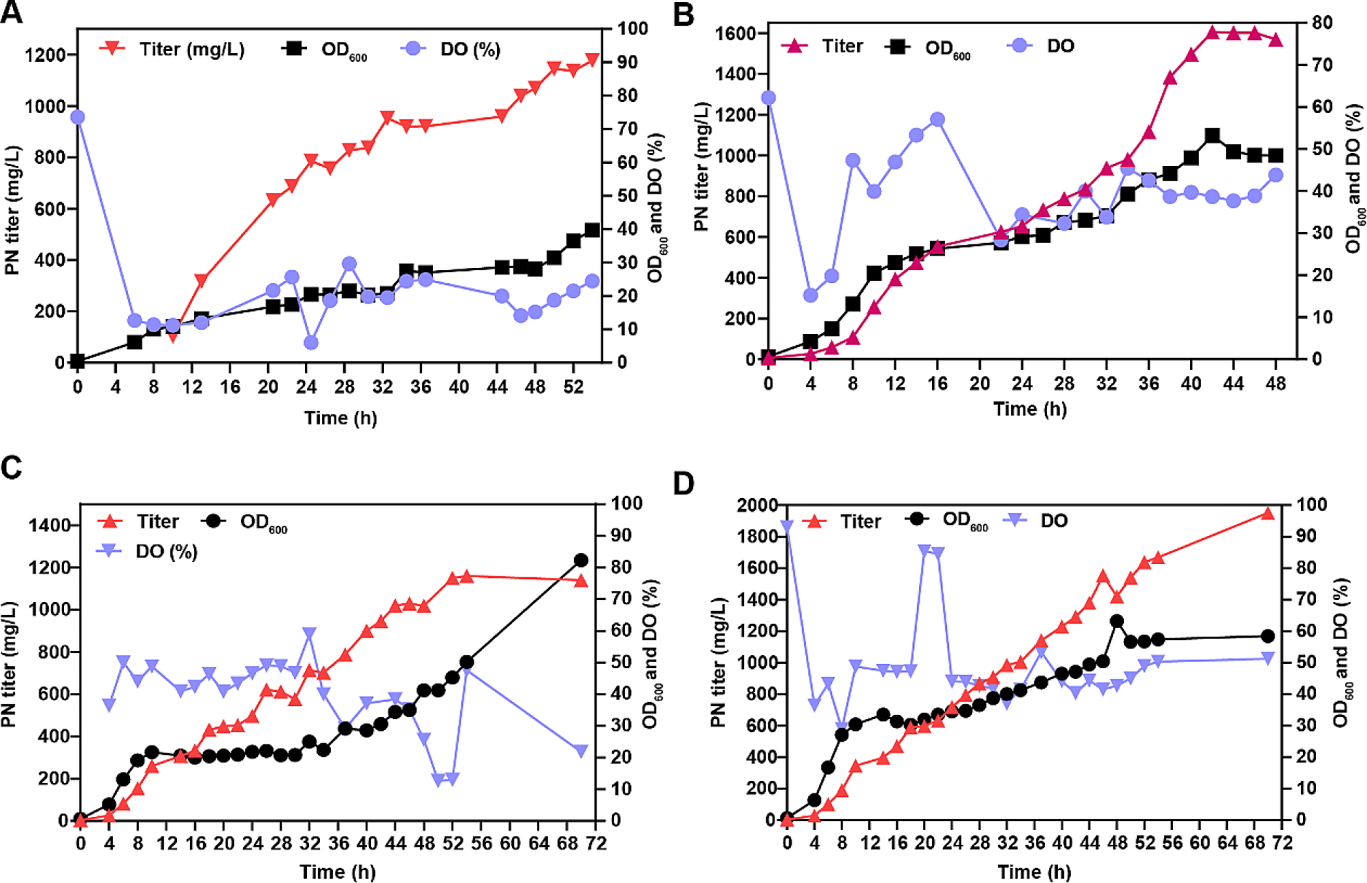
Time course of fed-batch fermentation under different conditions. **(A)** Time course of PN titer, OD_600_ and DO (%) of fed-batch fermentation I. **(B)** Time course of the pyridoxine titer, OD_600_ and DO (%) of fed-batch II. **(C)** and **(D)** Time course of the PN titer, OD_600_ and DO (%) of fed-batch III with different C/N ratios. **C**, C/N ratio = 6.76; **D**, C/N ratio = 4.56. The black line indicates the trend of the OD_600_, the red line shows the PN titer, and the purple line represents the DO (%)

In fed-batch II, the addition of 12 g/L succinate as a feeding strategy contributed to improved cell growth and PN production. Succinate serves as a supplementary carbon source during the fed-batch phase, supporting the metabolic activity of the strain. The DO level was increased and maintained at 40% to promote cell growth. PN started to be synthesized after cultivation for 4 h (Fig. 5B). Subsequently, PN was continuously produced, and the highest titer was measured to be 1.60 g/L after cultivation for 42 h, with an average productivity of 48.09 mg/L/h (Fig. 5B). However, the yield of PN did not continue to increase after the fermentation time exceeded 42 h. The OD_600_ increased to 50, surpassing the value observed in feeding batch I. This result suggested that the addition of succinic acid indeed promoted the growth of the strain.

Fed-batch III was used to investigate the effects of different C/N ratios in the culture medium on the yield during the fermentation process. The addition of 12 g/L succinate was used as the feeding medium for fed-batch II. The control group utilized the original medium with a C/N ratio of 6.76 (Fig. 5C), while the experimental group employed a modified medium with a lower C/N ratio of 4.56 (Fig. 5D). The results showed that fermentation with a lower C/N ratio balanced cell growth with production and that DO was more stable than fermentation with a C/N ratio of 6.76. The results revealed a state of slow and steady growth and production, which was the preferred state for PN production (Fig. 5D). After 48 h of fermentation, the control group yielded 1019 mg/L, while the experimental group with the optimized C/N ratio produced 1420 mg/L PN. As the fermentation continued and reached 70 h, the control group’s product yield increased to 1140 mg/L, whereas the experimental group achieved a higher final product yield of 1951 mg/L. These results indicate that the modified C/N ratio in the experimental group led to a significant improvement in product yield compared to that in the control group, even after an extended fermentation time. However, the average productivity was only 27.87 mg/L/h, which was lower than that of fed-batch II. Furthermore, there was a continued tendency for cell growth to increase, reaching approximately 60. This result further confirms the findings obtained from the transcriptomic and metabolic data. We also conducted tests on the plasmid stability of single-plasmid bacteria and observed that the plasmid retention rates at the end of fermentation were 97% and 98%, respectively (Fig. S5). It can be inferred that the single-plasmid strain is more stable than the dual-plasmid strain, based on the higher plasmid retention rates observed.

However, based on the relative yield comparison between the fed-batch fermentation and shake flask, it is clear that additional research is needed to optimize the control of the fermentation process. Additionally, it appears that there is a production limit for PN (1 to 1.5 g/L) in fed-batch fermentation within a 48-h period. Consequently, a thorough and systematic analysis of omics data is essential for identifying bottlenecks. This includes employing ionomics to ascertain the absence of crucial ions in the fermentation liquid or proteomics to determine the presence of toxic protein synthesis or inadequate enzyme synthesis within the vitamin B_6_ synthesis pathway, among other potential issues. Moreover, the limitations might also stem from the engineered strain itself. Switching chassis cells or boosting cell growth through metabolic engineering with omics data represents another potential strategy for increasing vitamin B_6_ production. We are committed to continuing our rigorous metabolic engineering and omics studies, aiming to increase fermentation yields and establish a microbial basis for the sustainable industrial production of vitamin B_6_.

## Conclusions

In this study, transcriptome and metabolome analyses of a high-yield strain provided insights into the possible inhibitory effect of PN accumulation on amino acids and compounds in the TCA cycle, particularly succinate and α-KG. The accumulation of PN may inhibit amino acid biosynthesis by interfering with enzyme activity, acting as a feedback inhibitor [48], disrupting amino acid balance [49], and affecting coenzyme availability [50] for essential metabolic processes. Afterward, a genetically modified single plasmid strain was utilized to assess different fermentation targets, such as specific amino acids, the C/N ratio (organic nitrogen source with high free amino acids), α-KG, and succinate. Ultimately, the engineered strain successfully reached a PN concentration of 514 mg/L in shake flasks. Moreover, through further optimization in a 5 L fed-batch fermentation process, an even higher production of 1951 mg/L was achieved. Overall, these results offer valuable insights and a scientific basis for future efforts aimed at enhancing PN yield through omics-guided approaches.

## Materials and methods

### Strains and growth conditions

The engineered *E. coli* strains used and constructed in this work are derivatives of the previous strain LL006. The strains and plasmids used are listed in Table 3. The primers used in this work are listed in Table S2. The *E. coli* DH5α strain was used for plasmid construction and replication. Lysogeny broth (LB) medium (10 g/L Bacto peptone, 5 g/L yeast extract, 10 g/L NaCl) was used to inoculate cells and propagate plasmids.

For shake-flask fermentation, activated colonies from the frozen stock were inoculated into tubes containing 5 mL of seed medium containing 10 g/L glycerol, 10 g/L Bacto peptone, 5 g/L yeast extract, and 5 g/L NaCl (pH adjusted to 6.5) containing the appropriate antibiotics. The bacterial culture was then inoculated at a concentration of 1% (v/v) into 30 mL of fermentation medium in a 250 mL shaker. Fermentation medium FM1.4 contained 15 g/L glycerol, 1 g/L glucose, 5 g/L yeast extract, 5 g/L Bacto peptone, 0.2 g/L MgSO_4_, 0.01 g/L MnSO_4_, 0.01 g/L FeSO_4_, and 100 mM Na_2_HPO_4_·12H_2_O, pH 6.5. FM1.5 medium contained 12 g/L glycerol, 4 g/L glucose, 8 g/L yeast extract, 7 g/L Bacto peptone, 2 g/L succinate, 2 g/L α-KG, 0.2 g/L MgSO_4_, 0.01 g/L MnSO_4_, 0.01 g/L FeSO_4_, and 100 mM Na_2_HPO_4_·12H_2_O, pH 6.5.

For bioreactor fermentation, activated colonies from the frozen stock were inoculated into 250 mL shakers containing 50 mL of seed medium. The composition of the seed medium was the same as above. After shaking for 8 h at 37 ℃, the culture was transferred to fermentation medium (1.4) at a dose of 10% (v/v). The feed contained 300 g/L glycerol, 20 g/L glucose, 5 g/L Bacto peptone, 5 g/L yeast extract, 6 g/L MgSO_4_·7H_2_O, 300 mg/L FeSO_4_·7H_2_O, 300 mg/L MnSO_4_·5H_2_O and 12 g/L succinate. All of the above were used in fed-batch I and II.

In fed-batch III, the new optimized medium was FM1.5, which included 12 g/L glycerol, 4 g/L glucose, 8 g/L yeast extract, 7 g/L Bacto peptone, 2 g/L succinate, 2 g/L α-KG, 0.1 M Na_2_HPO_4_·12H_2_O, 200 mg/L MgSO_4_·7H_2_O, 10 mg/L FeSO_4_, and 10 mg/L MnSO_4_ (pH = 6.5), while the feed contained 240 g/L glycerol, 80 g/L glucose, 7 g/L Bacto peptone, 8 g/L yeast extract, 6 g/L MgSO_4_·7H_2_O, 300 mg/L FeSO_4_·7H_2_O, 300 mg/L MnSO_4_·5H_2_O and 12 g/L succinate. Kanamycin (50 µg/mL) was added to the cultures when needed.

**Table 3:** Strains and plasmids used in this study

Strains	Genotype	Source
LL006	MG1655, *ΔpdxH*::*pdxST*-2, *Δpta*::P_tac_-*pdxP*(Eme)	Our Lab
LL388	LL006 harboring p15ASI-P_tac_-*epd* (Gni)-*pdxB* (Eco)-*dxs* (Eme)-P_J231119_ -*serC* (Eco), pRSFDuet-1_P3-*pdxA2*-*pdxJ1*	Our Lab
TZ01	LL006, *Δpta*::P3-*pdxA2*-*pdxJ1, ΔrpnD*::P3-*pdxA2*-*pdxJ1*	This study
TZ02	TZ01 harboring p15ASI-P_tac_-*epd* (Gni)-*pdxB* (Eco) -*dxs* (Eme)-P_J231119_ -*serC* (Eco)	This study
TZ03	TZ01 harboring pTrc99a-P_tac_-*epd* (Gni)-*pdxB* (Eco)-*dxs* (Eme)-P_J231119_ -*serC* (Eco)	This study
TZ04	TZ01 harboring pRSFDuet-1-P_tac_-*epd* (Gni)-*pdxB* (Eco) -*dxs* (Eme)-P_J231119_ -*serC* (Eco)	This study

### Genetic manipulation

All primers for PCR were synthesized by GENEWIZ Company (China), and PCR amplification was carried out using Primestar DNA polymerase from Takara Biomedical Technology (Beijing) Co., Ltd., following the manufacturer’s protocol. The P3-*pdxA2*-*pdxJ1* expression cassettes were amplified from the plasmid pRSFDuet-1_P3-*pdxA2*-*pdxJ1* and integrated into the *pta* and *rpnD* sites of LL006 using CRISPR/Cas9, respectively, resulting in strain TZ01. The correct strains were confirmed through PCR and DNA sequencing. To construct the expression plasmids, the P_tac_-*epd* (Gni)-*pdxB* (Eco)-*dxs* (Eme)-P_J231119_-*serC* (Eco) expression cassettes were cloned and inserted into pRSFDuet-1 and pTrc99a via Gibson assembly, resulting in the generation of pTrc99a-P_tac_-*epd* (Gni)-*pdxB* (Eco)-*dxs* (Eme)-P_J231119_-*serC* (Eco) and pRSFDuet-1-P_tac_-*epd* (Gni)-*pdxB* (Eco)-*dxs* (Eme)-P_J231119_-*serC* (Eco), respectively. Recombinant *E. coli* strains were created for the production of vitamin B_6_ by transforming the specified production strains with the corresponding plasmids.

### Bioinformatics analysis of transcriptome data

Cells for RNA-Seq were cultured in a 5 L bioreactor with 2 L of fermentation medium. After batch fermentation, 10 mL of cells were harvested at mid-log phase, flash-frozen in liquid nitrogen, and stored at -80 °C. Total RNA from each sample was extracted using TRIzol Reagent/RNeasy Mini Kit (Qiagen). Total RNA from each sample was quantified and qualified by Agilent 2100/2200 Bioanalyzer (Agilent Technologies, Palo Alto, CA, USA), NanoDrop (Thermo Fisher Scientific Inc.) and 1% agarose gel electrophoresis. Total RNA (1 µg) was used for subsequent library preparation. The rRNA was depleted from total RNA using an rRNA removal kit. The ribosomal-depleted RNA was then fragmented and reverse-transcribed. First-strand cDNA was synthesized using ProtoScript II Reverse Transcriptase with random primers and actinomycin D. Second-strand cDNA was synthesized using Second Strand Synthesis Enzyme Mix (including dACGTP/dUTP). The double-stranded cDNA purified by beads was then treated with End Prep Enzyme Mix to repair both ends, and a dA tail was added in one reaction, followed by T-A ligation to add adaptors to both ends. Selection of the size of the adaptor-ligated DNA was then performed using beads, and fragments of ∼ 400 bp (with an approximate insert size of 300 bp) were recovered. The dUTP-marked second strand was digested with Uracil-Specific Excision Reagent. Each sample was then amplified by PCR using the P5 and P7 primers, with both primers carrying sequences that can anneal with the flow cell to perform bridge PCR and the P5/P7 primer carrying the index allowing for multiplexing. The PCR products were cleaned using beads, validated using a Qsep100 (Bioptic, Taiwan, China), and quantified by Qubit3.0 Fluorometer (Invitrogen, Carlsbad, CA, USA). Then, libraries with different indices were multiplexed and loaded on an Illumina HiSeq/Novaseq instrument according to the manufacturer’s instructions (Illumina, San Diego, CA, USA) or an MGI2000 instrument according to the manufacturer’s instructions (MGI, Shenzhen, China). Sequencing was carried out using a 2 × 150 paired-end (PE) configuration; image analysis and base calling were conducted by HiSeq Control Software (HCS) + OLB + GAPipeline-1.6 (Illumina) on the HiSeq instrument, NovaSeq Control Software (NCS) + OLB + GAPipeline-1.6 (Illumina) on the NovaSeq instrument, and Zebeacall on the MGI2000 instrument.

For the data analysis, to remove technical sequences, including adapters, polymerase chain reaction (PCR) primers, fragments thereof, and bases with a quality lower than 20, pass filter data in fastq format were processed by Cutadapt (version 1.9.1, phred cutoff: 20, error rate: 0.1, adapter overlap: 1 bp, min. length: 75, proportion of N: 0.1). First, reference genome sequences and gene model annotation files of related species were downloaded from genome websites, such as UCSC, NCBI, and ENSEMBL. Second, Bowtie2 (v2.2.6) was used to index the reference genome sequence. Finally, the clean data were aligned to the reference genome via Bowtie2 software (v2.2.6). Initially, transcripts in fasta format were converted from known gff annotation files and indexed properly. Then, with the file as a reference gene file, HTSeq (v0.6.1p1) was used to estimate gene expression levels from the paired-end clean data. Differential expression analysis was performed with the DESeq2 Bioconductor package, a model based on a negative binomial distribution. After adjusting Benjamini and Hochberg’s approach for controlling the false discovery rate, the Padj of genes was set to < 0.05 to detect DEGs. Gene Ontology (GO) terms in GOSeq (v1.34.1) were used to annotate a list of enriched genes with a p value less than 0.05. In addition, topGO was used to plot the DAG. Rockhopper uses a Bayesian approach to create a transcriptome map including transcription start/stop sites for protein-coding genes and novel transcripts identified by Rockhopper. Samtools v0.1.19 with the command mpileup and Bcftools v0.1.19 + were used for SNV calling. Rockhopper (v2.0.3) was used to predict operons, transcription start sites (TSSs) and transcription stop sites (TTSs). RBSfinder (v1.0) was used for SD sequence prediction. TransTermHP (v2.09) can accurately detect Rho-independent transcription terminators. The novel intergenic transcripts were subjected to BLAST searches against the NR database, and nonannotated transcripts were considered potential trans-encoded sRNAs. The novel antisense transcript was treated as a cis-encoded sRNA. The secondary structures of the sRNAs were predicted using RNAfold (2.3.2).

### Samples for metabolomic analysis

To monitor changes in intracellular metabolite content, samples were collected from the cultures at 6, 16, 36 and 42 h. Each sample consisted of 0.2 mL, which was immediately mixed thoroughly with 1 mL of precooled methanol (40% concentration) at -20 ℃ by vortexing for one second. Three parallel samples were prepared within one minute. Subsequently, the samples were centrifuged at a speed of 10,625 × g and a temperature of 4 ℃ for two minutes to remove the supernatant while preserving the bacterial cells at -80 ℃. The cells were then individually treated with precooled 40% methanol (2 mL) at -20 ℃ and centrifuged for two minutes. The resulting pellets were resuspended in an acidic acetonitrile-water solution (1:1 v/v) containing formic acid (0.1%) that had been precooled to -20 ℃ as well as an ethanol-water solution (3:1 v/v) heated to 100 ℃. The extraction and derivatization procedures followed the protocol described by Chang et al. [51–53], after which the obtained supernatant was freeze-dried and stored at -80 °C.

Intracellular metabolites were analyzed using an ultra-performance liquid chromatography (UPLC) system (Nexera 30 A, Shimadzu, Kyoto, Japan) coupled with a mass spectrometer (TripleTOF™ 5600, Applied Biosystem Sciex, United States) in negative electrospray ionization (ESI) mode. Most of the metabolites were separated by LC equipped with a SeQuant ZIC-HILIC column (100 × 2.1 mm, 3.5 μm, Merck, Germany). The mobile phase was composed of a gradient comprising 10 mM ammonium acetate (A) and 100% acetonitrile (B) as follows: 0–3 min, 90% B; 3–6 min, 90–60% B; 6–25 min, 60–50% B; 25–30 min, 50% B; 30–30.5 min, 50–90% B; and 30.5–38 min, 90% B, at a flow rate of 0.2 mL/min. The relative content of metabolites was normalized to the cell density (OD_600_).

### Fed-batch fermentation

After overnight incubation in a seed medium at 37 ℃, the seed culture (10%, v/v) was inoculated into a 5 L bioreactor containing 2 L of fermentation medium supplemented with 1 L of conventional feed. The primary focus is on promoting cell growth while maintaining dissolved oxygen (DO) levels above 40% through regulation of the agitation rate (200–400 rpm). It is assumed that the uninoculated bioreactor achieves maximum dissolved oxygen at an agitation rate of 200 rpm. A continuous supply of conventional feed is introduced to sustain dissolved oxygen levels above 40%. Throughout the fermentation process, circulating water is utilized to maintain a constant temperature of 37 °C. Additionally, pH control is achieved by incorporating ammonium hydroxide and phosphoric acid.

### Analysis of pyridoxine and organic acid by HPLC

The cell density (OD_600_) was measured using a Hybrid Multi-Mode Reader (Synergy Neo2, Bio Tek, USA). PN quantification was performed using a Thermo Fisher high-performance liquid chromatography (HPLC) system (UltiMate™ 3000) equipped with an FLD-3400 detector and employing a gradient program based on previously published protocols with minor modifications. In brief, the fermentation mixture underwent centrifugation, and the resulting supernatant was subjected to HPLC analysis utilizing fluorescence detection. The excitation and emission wavelengths were set at 293 and 395 nm, respectively. The separation of PN was achieved using an octadecylsilyl (ODS) column (Cosmosil AR-II; 250 by 4.6 mm, particle size: 5 μm; Nacalai Tesque) through a gradient program. Mobile phase A consisted of 33 mM phosphoric acid and 8 mM 1-octanesulfonic acid adjusted to pH 2.4 with KOH, while mobile phase B consisted of acetonitrile at an 80% (v/v) concentration. The total flow rate used for separation was maintained at 0.8 mL/min. The linear gradient program employed in this study involved the following steps: from 0% mobile phase B to 1% B over 5 min, from 1% B to 19% B over 5 min, from 19% B to 28% B over 10 min, from 28% B to 63% B over 5 min, from 63% B to 0% B over 2 min, and finally maintaining the composition as 0% B for another 3 min. All the data are presented as the means ± SDs of three independent replicates. The software Origin Pro (version 9.1) or GraphPad Prism (version 8) was utilized for analyzing fermentation data.

The quantitative analysis of the organic acids was carried out using an Agilent 1260 Infinity series HPLC system (Agilent Technologies, Palo Alto, California, USA), which consisted of a quaternary pump, a thermostated automated injector, a thermostated column compartment and a refractive index detector. The samples were separated and analyzed by an Aminex® HPX-87 H Ion Exclusion Column (BIO-RAD, 7.8 mm × 300 mm). The mobile phase was composed of 5 mM H_2_SO_4_ in water (eluent A) using gradient elution as follows: 0–30 min, 100% of A. The injection volume, flow rate and ambient temperature were 20 µL, 0.5 mL/min and 60 ℃, respectively. The RI detection was set as follows: zero compensation, 5%; attenuation, 500,000 nRIU.

### Plasmid stability analysis

The fermentation samples in the bioreactor were plated on nonselective plates and incubated at 37 °C for 16–20 h. A single colony was randomly picked and spotted on an LB plate supplemented with kanamycin. The colonies were counted, and the percentage stability was calculated by determining the ratio of the number of colonies on plates with growth to the total number of spots.

## Electronic supplementary material

Below is the link to the electronic supplementary material.

Supplementary Material 1

## Data Availability

No datasets were generated or analysed during the current study.

## Abbreviations

ICDH: isocitrate dehydrogenase
DXP: 1-deoxy-D-xylulose-5-phosphate
Glycerol-3P: glycerol-3-phosphate
DAHP: 3-deoxy-D-arabinoheptulosonate-7-phosphate
G6P: glucose-6-phosphate
F6P: fructose-6-phosphate
F1.6BP: fructose-1,6-bisphosphate
G3P: glyceraldehyde-3-phosphate
1,3-BPG: 1,3-bisphosphoglyceride
3-PG: 3-phosphoglycerate
2-PG: 2-phosphoglycerate
PEP: phosphoenolpyruvate
PYR: pyruvate
6PGNL: 6-phosphogluconolactone
6PG: 6-phosphogluconate
Ru5P: ribulose 5-phosphate
PRPP: phosphoribosyl pyrophosphate
Xu5P: xylulose 5-phosphate
R5P: ribose 5-phosphate
S7P: sedoheptulose 7-phosphate
E4P: erythrose 4-phosphate
OAA: oxaloacetic acid
His: histidine
Trp: tryptophan
Ser: serine
Gly: glycine
Val: valine
Ile: isoleucine
Leu: leucine
Asp: aspartate
Hse: homoserine
Met: methionine
Thr: threonine
Glu: glutamate
Gln: glutamine
GlpK: glycerol kinase
HisA: 1-(5-phosphoribosyl)-5-[(5-phosphoribosylamino) methylideneamino] imidazole-4-carboxamide isomerase
HisB: imidazoleglycerol-phosphate dehydratase/histidinol-phosphatase
HisC: histidinol-phosphate aminotransferase
HisF: imidazole glycerol phosphate synthase subunit
HisG: ATP phosphoribosyltransferase
HisH: imidazole glycerol phosphate synthase subunit
HisI: putative bifunctional phosphoribosyl-AMP cyclohydrolase/phosphoribosyl-ATP pyrophosphatase
FbaA: fructose-bisphosphate aldolase class II
GapA: glyceraldehyde-3-phosphate dehydrogenase A
Pgk: phosphoglycerate kinase
PcK: phosphoenolpyruvate carboxykinase (ATP)
GltA: citrate synthase
GlcB: malate synthase G
AceA: isocitrate lyase
SdhA: succinate quinone oxidoreductase, FAD binding protein
SdhC/SdhD: succinate quinone oxidoreductase, membrane protein
FrdA: fumarate reductase flavoprotein subunit
SucA: subunit of E1(0) component of 2-oxoglutarate dehydrogenase
SucB: dihydrolipoyltranssuccinylase
SucC: succinyl-CoA synthetase subunit beta
SucD: succinyl-CoA synthetase subunit alpha
TktB: transketolase 2
TalA: transaldolase A
TrpD/E: anthranilate synthase subunit
GlyA: serine hydroxymethyltransferase
IlvN/B: acetohydroxy acid synthase I subunit
IlvC: ketol-acid reductoisomerase (NADP^+^)
IlvD: dihydroxy-acid dehydratase
AspC: aspartate aminotransferase
ThrA: fused aspartate kinase/homoserine dehydrogenase 1
ThrB: homoserine kinase
ThrC: threonine synthase
MetC: cystathionine beta-lyase/L-cysteine desulfhydrase/alanine racemase
MetE: cobalamin-independent homocysteine transmethylase
MetF: 5,10-methylenetetrahydrofolate reductase
GltB/D: glutamate synthase subunit
GlnA: glutamine synthetase
GadA/B: glutamate decarboxylase A/B
GlsA: glutaminase 1
GuaA: GMP synthetase
CarA: carbamoyl-phosphate synthetase small subunit
4HTP: 4-phosphohydroxy-L-threonine
