# Correction: Enhancement of vitamin B6 production driven by omics analysis combined with fermentation optimization

**DOI:** 10.1186/s12934-024-02532-9

**Published:** 2024-09-28

**Authors:** Zhizhong Tian, Linxia Liu, Lijuan Wu, Zixuan Yang, Yahui Zhang, Liping Du, Dawei Zhang

**Affiliations:** 1 College of Biotechnology, Tianjin University of Science and Technology, Tianjin, 300457 China; 2 Tianjin Institute of Industrial Biotechnology, Chinese Academy of Sciences, Tianjin, 300308 China; 3 National Center of Technology Innovation for Synthetic Biology, Tianjin, 300308 China; 4 Key Laboratory of Engineering Biology for Low-Carbon Manufacturing, Tianjin Institute of Industrial Biotechnology, Chinese Academy of Sciences, Tianjin, 300308 China; 5 University of Chinese Academy of Sciences, Beijing, 100049 China

**Correction: Microbial cell factories (2024) 23: 137**

10.1186/s12934-024-02405-1

The Data availability statement should read:

The raw transcriptome and metabolome data have been deposited in Zenodo 10.5281/Zenodo.13777074

