# Supplementary material for: Enhancement of vitamin B6 production driven by omics analysis combined with fermentation optimization
Source: Microb Cell Fact. 2024 May 15;23:137. doi: 10.1186/s12934-024-02405-1 (PMC11095007; doi:10.1186/s12934-024-02405-1)
Supplement: Supplementary file 1 — Supplementary Material 1 [file 12934_2024_2405_MOESM1_ESM.docx]

**Supplementary Information**

Enhancement of pyridoxine production driven by omics analysis combined with fermentation optimization

**Authors：**

Zhizhong Tian^1,2#^, Linxia Liu^2,3,4#^, Lijuan Wu^2^, Zixuan Yang^2^, Yahui Zhang^2^, Liping Du^1*^, Dawei Zhang^2,3,4,5*^

**Affiliations:**

^1^ School of Biological Engineering, Tianjin University of Science and Technology, Tianjin, China.

^2^ Tianjin Institute of Industrial Biotechnology, Chinese Academy of Sciences, Tianjin, China.

^3^ National Center of Technology Innovation for Synthetic Biology, Tianjin, China.

^4^ Key Laboratory of Engineering Biology for Low-Carbon Manufacturing, Tianjin Institute of Industrial Biotechnology, Chinese Academy of Sciences, Tianjin, China.

^5^ University of Chinese Academy of Sciences, Beijing, China.

#Equal Contributions


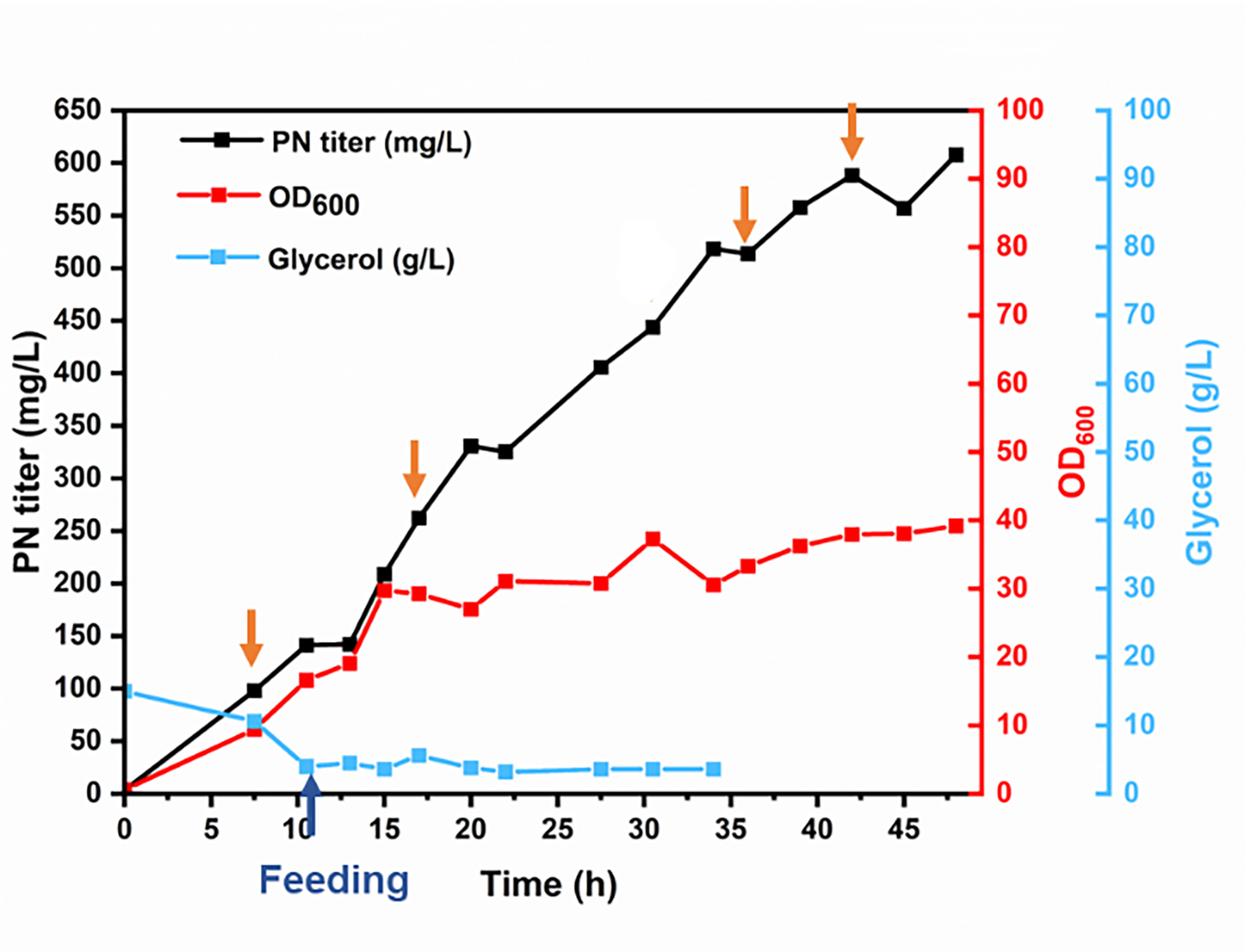


**Supplementary figure S1.** Time course of PN titer, OD_600_ and glycerol of fed-batch fermentation of LL388. The blue arrow denotes the initiation of feeding, while the brown arrow signifies the time point for sample collection (6, 16, 36, and 42 h).

**
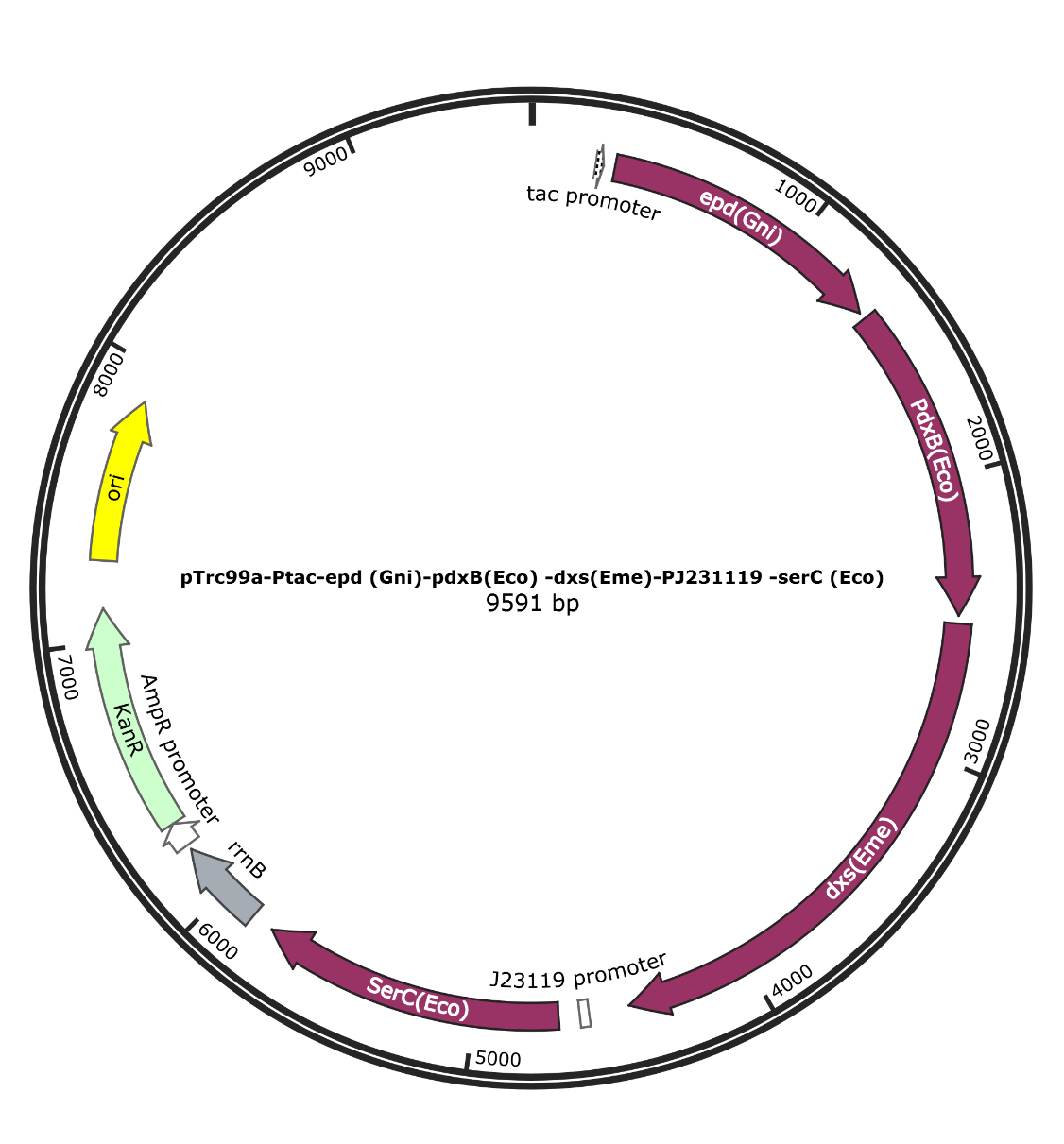
**

**Supplementary figure S2.** The plasmid map of pTrc99a-P_tac_-*epd* (Gni)-*pdxB* (Eco) -*dxs* (Eme)-P_J231119_ -*serC* (Eco).

**Supplementary figure S3.** The product synthesis rate and the specific growth rate of fed-batch Ⅰ**.**

**Supplementary figure S4.** The intracellular concentration of succinate, acetate, and pyruvate changed with time. The orange line, blue line and black line represent succinic acid, acetic acid and pyruvic acid, respectively.


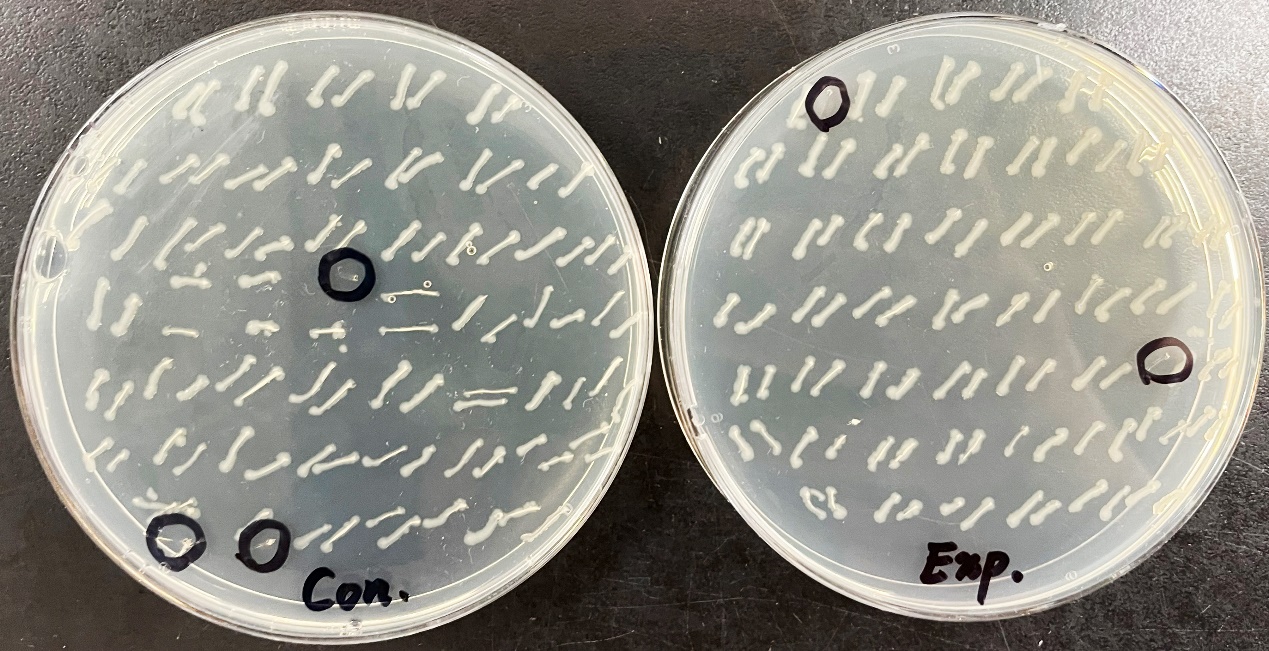


**Supplementary figure S5.** The plasmid stability of fed-batch Ⅲ after 70 h fermentation. Con. the control group with original medium; Exp. the experimental group with the optimized C/N ratio.

Table S1. Effect estimates for PN production from the result of Plackett-Burman design

| **Amino Acids^1^** | **Effect** | **S.E.^2^** | **T value** | **P-value** | **Confidence level (%)^3^** |
| --- | --- | --- | --- | --- | --- |
| Gly | 33.3 | 24.9 | 0.67 | 0.624 | 37.6 |
| His | -35.4 | 24.9 | -0.71 | 0.606 | 39.4 |
| Val | -22.6 | 24.9 | -0.45 | 0.728 | 27.2 |
| Asp | -46.3 | 24.9 | -0.93 | 0.523 | 47.7 |
| Thr | 42.2 | 24.9 | 0.85 | 0.552 | 44.8 |
| Met | -33.4 | 24.9 | -0.67 | 0.624 | 37.6 |
| Ile | -23.0 | 24.9 | -0.46 | 0.724 | 27.6 |
| Glu | 16.5 | 24.9 | 0.33 | 0.796 | 20.4 |
| Arg | -35.2 | 24.9 | -0.71 | 0.608 | 39.2 |
| Trp | 3.6 | 24.9 | 0.07 | 0.954 | 4.6 |

^1^Amino Acids: Gly glycine; His histidine; Val valine; Asp aspartic acid; Thr: threonine; Met methionine; Ile isoleucine; Glu glutamic acid; Arg arginine; Trp tryptophan.

^2^S.E.: Standard Error.

^3^Confidence level: The percentage of all possible samples that are expected to include the true population parameter.

Table S2. primers used in this study

| Name | Sequences (5’-3’) |
| --- | --- |
| Pta-A2J1-F | CAACGCTGACTGGATCGAATCTCTGACGCGACTCCTGCATTATACCTAGGACAG |
| Pta-A2J1-R | GATCAACGATTTCAATCCCTGCACCCAGGCGAAAAAACCCCGCCGAAGCGGGGT |
| Pta-UP-F | AACAAGCAGAAAATATTATTCATTTATTTACGTTGACGA |
| Pta-UP-R | GCTAAGATCTGACTCCATAACAGAGTACTCG |
| Pta-DN-F | GTTTTAGAGCTAGAAATAGCAAGTTAAAATAAGGCT |
| Pta-DN-R | CTCTTGTATCTATCAGTGAAGCATCAAGACT |
| Pta-V1-F | GCAAACGTATCGTACTGCCGGAGTTTTAGAGCTAGAAATAGCAAGTTAAAATAAGGCTAGTC |
| Pta-V1-R | CTAGCTGTCCTAGGTATAATGCAGGAGTCGCGTCAGAGATTCGATCCAGTCAGCG |
| Pta-V2-F | CGCAAAAAACCCCGCTTCGGCGGGGTTTTTTCGCCTGGGTGCAGGGATTGAAATCGTTG |
| Pta-V2-R | CTAAAACTCCGGCAGTACGATACGTTTGCTAAGATCTGACTCCATAACAGAGTACTC |
| RpnD-A2J1-F | ATCGAAGAATGCGCTGCTCCCCTTTTAGCGACTCCTGCATTATACCTAGGACAG |
| RpnD-A2J1-R | CCGCACGGAAGCTTTATAACGCCGCGTTGCGAAAAAACCCCGCCGAAGCGGGGT |
| RpnD-UP-F | ACAGACGAAGAATCCATGGGCCTGTCCTGAATTAAGCAAAGTACGCTTTGTTC |
| RpnD-UP-R | CTGTCCTAGGTATAATGCAGGAGTCGCTAAAAGGGGAGCAGCGCATTCTTCGAT |
| RpnD-DN-F | AAAAACCCCGCTTCGGCGGGGTTTTTTCGCAACGCGGCGTTATAAAGCTTCC |
| RpnD-DN-R | TATTGGTGAGAATCCAAGCTTCCATTCAGTCGATGAAAAATTGCGGGCGCTA |
| RpnD-V1-F | TGAATGGAAGCTTGGATTCTCACC |
| RpnD-V1-R | TAAGATCTGACTCCATAACAGAGTACTC |
| RpnD-V2-F | GCAATTTAACAGCGCAGTTATCGTTTTAGAGCTAGAAATAGCAAGTTAAAATAAGGCTAGTC |
| RpnD-V2-R | ACAGGCCCATGGATTCTTCGTC |
| V-A2J1-test-F | ATGGCACATAGCCTTGCTCAAAT |
| V-A2J1-test-R | GGATTTGTTCAGAACGCTCGGTT |
| pTrc-F | GCTGTTTTGGCGGATGAGAG |
| pTrc-R | CAGCTCATTTCAGAATATTTGCCAGAACC |
| pRSF-F | CGCAAAAAACCCCGCTTCG |
| pRSF-R | ATTTCCTAATGCAGGAGTCGCATAAG |
| pTrc-R90-F | CATAACGGTTCTGGCAAATATTCTGAAATGAGCTGGGATCTCGACGCTCTCCCTTATG |
| pTrc-R90-R | TGAAAATCTTCTCTCATCCGCCAAAACAGCTTAACCGTGACGGCGTTCGAAC |
| pRSF-R90-F | CTCCCTTATGCGACTCCTGCATTAGGAAATGGATCTCGACGCTCTCCCTTATG |
| pRSF-R90-R | AAAAACCCCGCCGAAGCGGGGTTTTTTGCGTTAACCGTGACGGCGTTCGAAC |
| pRSF-R90-test-F | GCTATCATGCCATACCGCGA |
| pRSF-R90-test-R | CGGTGCGAACACATAATGCC |
| pTrc-R90-test-F | CGCACTCCCGTTCTGGATAAT |
| pTrc-R90-test-R | TAACGGTCTCTGCGGTGGTA |
